# Classification of Sparkling Wine Style and Quality by MIR Spectroscopy

**DOI:** 10.3390/molecules20058341

**Published:** 2015-05-08

**Authors:** Julie Culbert, Daniel Cozzolino, Renata Ristic, Kerry Wilkinson

**Affiliations:** School of Agriculture, Food and Wine, University of Adelaide, PMB 1, Glen Osmond, SA 5064, Australia; E-Mails: julie.culbert@adelaide.edu.au (J.C.); daniel.cozzolino@adelaide.edu.au (D.C.); renata.ristic@adelaide.edu.au (R.R.)

**Keywords:** sparkling wine, mid-infrared spectroscopy, principal component analysis

## Abstract

In this study, the suitability of attenuated total reflection (ATR) mid-infrared (MIR) spectroscopy, combined with principal component analysis (PCA) and partial least squares (PLS) regression, was evaluated as a rapid analytical technique for the classification of sparkling wine style and quality. Australian sparkling wines (*n* = 139) comprising a range of styles (*i.e.*, white, rosé, red, Prosecco and Moscato) were analyzed by ATR-MIR spectroscopy combined with multivariate data analysis. The MIR spectra of 50 sparkling white wines, produced according to four different production methods (*i.e.*, Carbonation, Charmat, Transfer and Methodé Traditionelle) were also evaluated against: (i) quality ratings determined by an expert panel; and (ii) sensory attributes rated by a trained sensory panel. Wine pH, titratable acidity (TA), residual sugar (RS), alcohol and total phenolic content were also determined. The sparkling wine styles were separated on the PCA score plot based on their MIR spectral data; while the sparkling white wines showed separation based on production method, which strongly influenced the style and sensory properties of wine (*i.e.*, the intensity of fruit *versus* yeast-derived characters). PLS calibrations of 0.73, 0.77, 0.82 and 0.86 were obtained for *sweetness*, *tropical fruit*, *confectionary* and *toasty* characters (on the palate), respectively.

## 1. Introduction

Sparkling wine represents a small but significant proportion (~10%, 37 ML in 2012) of the Australian wine industry’s total production [1]. The Australian sparkling wine market comprises white and rosé Moscato, and white, rosé and red sparkling wines, both as mono-varietals and blends, at commercial, premium and luxury price points. Furthermore, the style and quality of sparkling white wine can vary greatly depending on the method of production. The most basic method (*i.e.*, carbonation) involves carbon dioxide gas being infused (‘bubbled’) directly into the wine, but carbon dioxide can also be introduced during secondary fermentation; either in tanks (*i.e.*, Charmat) or in bottles (*i.e.*, Transfer or Methodé Traditionelle) [2]. In the transfer method, the individual bottles of sparkling wine are blended in a pressurized tank following secondary fermentation, before being re-bottled; whereas the Methodé Traditionelle involves individual bottles being disgorged (*i.e.*, yeast sediment is removed), dosage is then added and the bottle is packaged (corked and labelled), such that the bottle purchased by the consumer is the same bottle that was used for secondary fermentation [2].

MIR spectroscopy is a valuable analytical tool that measures changes in the absorption of energy by different functional groups, for example those comprising carbon-oxygen (C–O), oxygen-hydrogen (O–H) and carbon-carbon (C–C) bonds, due to characteristic vibrational frequencies associated with stretching and/or bending of bonds [3]. Unique spectral fingerprints can therefore be obtained for different samples, with more absorption bands typically being observed for more complex molecular structures [4,5]. Recent advances in infrared spectroscopic instrumentation, together with the introduction of sampling techniques such as attenuated total reflection (ATR), have made spectral methods increasingly attractive for the rapid analysis of complex matrices across many scientific fields. ATR-MIR requires minimal sample preparation, so samples can be analyzed quickly, non-destructively and at low cost [6] and compared to spectra in the near infrared region (14,000 to 4000 cm^−1^), the MIR region (4000 to 400 cm^−1^) typically comprises more spectral peaks, which are better defined and therefore, more easily interpreted [7]. As such, MIR spectroscopy has become a popular technique for qualitative analysis.

Wine is a complex matrix, with diverse compositional and sensory characteristics. The vast array of volatile and non-volatile constituents that occur in wine at concentrations ranging from the low ng/L to the low g/L, can therefore make analysis of wine chemistry quite challenging. However, MIR spectroscopy has increasingly been employed for the rapid, convenient analysis of both juice and wine. For example, routine analysis of total soluble solids (TSS), pH, total phenolics, ammonia, free amino nitrogen (FAN) and yeast assimilable nitrogen (YAN) has been performed simultaneously on grape juice using MIR spectroscopy [8]; while MIR-based techniques have also been used to differentiate wines based on variety [9,10] or geographical origin [11,12,13], to identify wines tainted by bushfire smoke [14]; and even to validate the authenticity of wine [5,15]. This study sought to determine whether or not ATR-MIR spectroscopy can be used as a rapid analytical tool for the classification of Australian sparkling wines, according to style and quality.

## 2. Results and Discussion

### 2.1. Classification of Sparkling Wine Style by MIR Spectroscopy

**Figure 1:**
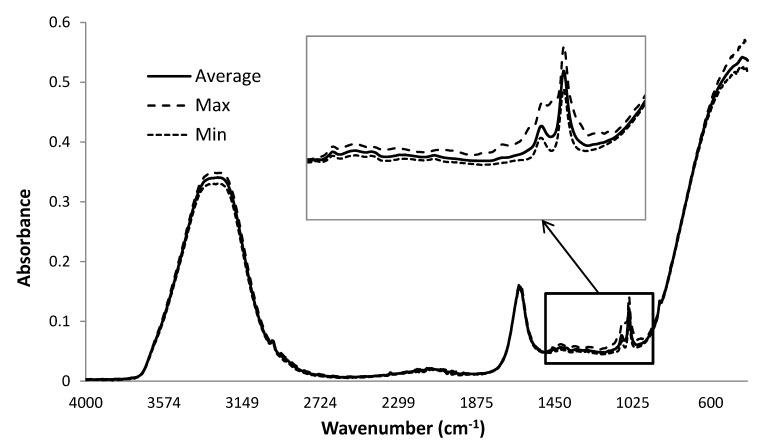
Mean, maximum and minimum ATR-MIR spectra (4000–400 cm^−1^) obtained from (degassed) sparkling wine samples (*n* = 139).

The ATR-MIR spectra of commercial sparkling wines showed moderate to strong absorbance peaks at 1045, 1085, 1640 and 3300 cm^−1^ (Figure 1); with peaks at 3300 and 1640 cm^−1^ corresponding to the O–H stretching and bending respectively, associated with water [16,17]. The MIR region between 1100 and 1000 cm^−1^ has previously been attributed to C–O vibrations of sugars, such as glucose and fructose, and alcohols, phenols, esters and lactones [3]. In particular, absorbance in the region of 1080 to 1045 cm^−1^ has been associated with C–OH bonds present in primary alcohols (e.g., ethanol), glycerol and sugars (glucose and fructose) [4,11,13,15]; *i.e.*, compounds which are likely to be constituents of sparkling wine.

A comparison of the minimum and maximum ATR-MIR spectra (Figure 1) obtained from the (degassed) sparkling wine samples indicated most of the variation observed amongst the samples occurred within the ‘fingerprint’ region; *i.e.*, between 1500 and 900 cm^−1^. For grape and wine samples, this region is known to contain absorbance bands attributable to water, sugars and phenolic compounds [8], and results from stretching and/or bending of CH–OH, C–C, C–O and C–H bonds. Multivariate analysis was therefore performed on the MIR ‘fingerprint’, given this region accounted for the most variation.

**Figure 2:**
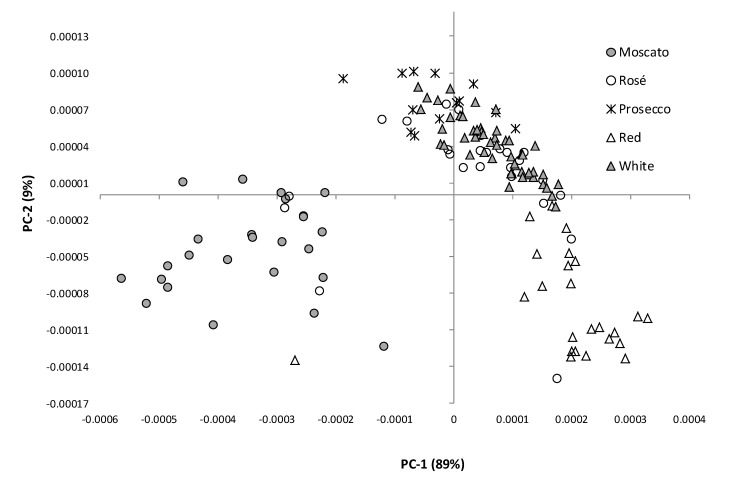
Score plot of the first two PC’s derived from the MIR ‘fingerprint’ (1500–900 cm^−1^) of white, rosé, red, Prosecco and Moscato wines (*n* = 50, 25, 25, 14 and 25, respectively).

The PCA score plot of the first two principal components (PC) derived from the ATR-MIR ‘fingerprint’ spectra of all sparkling wine samples is shown in Figure 2. The first principal component (PC) explains 89% of the variation observed and resulted in clear separation of Moscato wines (lower left quadrant) from the other sparkling wine styles. Separation of sparkling red (lower right quadrant) and sparkling white, rosé and Prosecco wines (upper quadrants) was also observed. Several outliers were observed, *i.e.*, individual wines that did not cluster with other wines of the same style, namely: three sparkling rosé wines and a sparkling red wine, that instead clustered amongst the Moscato wines; and two sparkling rosé wines that instead clustered together with the remaining sparkling red wines. A plausible explanation for these outliers is suggested below.

**Figure 3:**
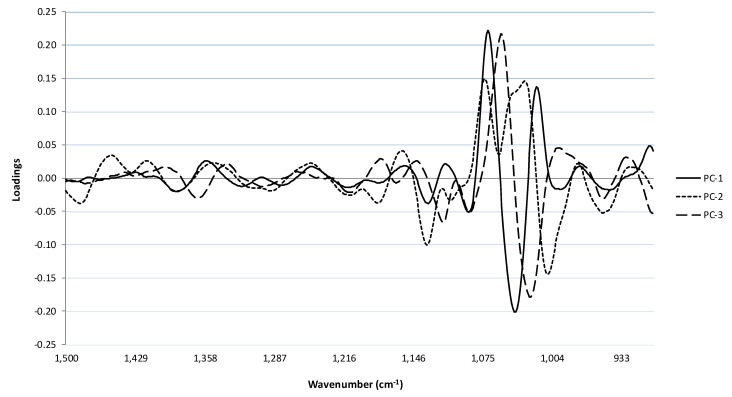
Loadings for the first three principal components for the fingerprint region derived from the MIR spectra of the sparkling wines.

The clustering pattern of sparkling wines likely reflects compositional differences that can be attributed to both varietal expression and wine style. In terms of grape variety: sparkling red wines largely comprised Shiraz (or blends thereof); sparkling white wines comprised the classic varieties, *i.e.*, Chardonnay, Pinot Noir and/or Pinot Meunier; sparkling rosé wines were predominantly Pinot Noir (or blends thereof); and Moscato comprised Muscat varieties. Analysis of several basic wine chemistry parameters, *i.e.*, pH, titratable acidity (TA), residual sugar (RS), alcohol content and total phenolics (Table 1), demonstrated several large compositional differences between the various sparkling wine styles; primarily related to residual sugar, alcohol and phenolic content. As expected, Moscato wines had the highest residual sugar levels (91 g/L on average, compared with 10 to 30 g/L for the other sparkling wines) and the lowest alcohol levels (*i.e.*, 7%, compared with 11% to 13%). Sparkling red wines typically had higher alcohol levels, and the highest total phenolics (due to the presence of grape skins during primary fermentation). To determine to what extent these constituents influenced wine clustering in the PCA score plot, the loadings for the first three PCs derived from the fingerprint region of sparkling wine MIR spectra were evaluated (Figure 3).

**Table 1:** Composition of the different styles of sparkling wine studied.

Sparkling Wine Style		pH	TA (g/L)	RS (g/L)	Alcohol (% abv)	Total Phenolics (au)
White (*n* = 50)	range mean	3.0–3.5 3.2	5.8–9.6 7.5	0.5–20.1 11.2	10.3–13.1 11.9	0.3–5.8 2.9
Rosé (*n* = 25)	range mean	3.1–3.5 3.3	5.3–8.4 6.8	5.1–86.7 22.9	7.9–13.7 11.6	2.2–6.2 4.0
Red (*n* = 25)	range mean	3.4–3.9 3.5	5.1–7.5 6.3	7.2–117.6 32.7	8.4–15.0 13.4	37.1–67.0 49.9
Prosecco (*n* = 14)	range mean	2.9–3.5 3.2	5.6–7.8 6.4	0.4–22.4 10.6	9.2–12.2 11.0	0.0–3.3 0.9
Moscato (*n* = 25)	range mean	3.0–3.5 3.2	4.9–9.0 6.7	57.9–143.1 90.5	5.1–9.9 7.4	0.8–15.5 4.6

The highest loadings for PC-1 were observed at 1069, 1040 and 1020 cm^−1^. As indicated above, absorbance in the region of 1080 to 1045 cm^−1^ is usually associated with the C–C and C–OH bonds of primary alcohols (e.g., ethanol), glycerol and sugars [4,5,11,13,14,15]. This suggests the variation observed between samples in the first principal component is largely explained by differences in residual sugar and alcohol content. For PC-2, which explained 9% of variation, the highest loadings were also in this region (*i.e.*, at 1073 to 1008 cm^−1^). Less significant, but larger loadings were also observed for PC-2 within the 1500 to 1400 cm^−1^ region, which may be indicative of aromatic C–C stretching [14] and/or absorbance by CO=O, C=C, C–H_2_ and C–H_3_ bonds from organic acids and aldehydes [11,13,15]. PC-2 loadings were also observed at 1130 and 1150 cm^−1^, with the latter possibly being characteristic of pyranose sugars [16]. Therefore, in addition to sugar and alcohol, phenolics and organic acids may also have contributed to the clustering patterns of the different sparkling wines.

Consideration of the basic chemical parameters of individual sparkling wines also helped to explain the clustering of wines identified (above) as outliers. The three sparkling rosé wines and the sparkling red wine that clustered amongst the Moscato wines were found to contain high residual sugar levels; between 63 and 87 g/L, and 118 g/L, for the sparkling rosé and sparkling red wines, respectively. Of the two remaining sparkling rosé wines that were clustered amongst the sparkling red wines, one also had high residual sugar (*i.e.*, 60 g/L), and both had unusually high alcohol levels (*i.e.*, 13.4% and 13.7%). The residual sugar and/or alcohol content of these sparkling wines may therefore have more strongly influenced their clustering, than varietal expression. Collectively, these observations highlight the influence of sugar and alcohol on the positioning of individual sparkling wines on the PCA score plot.

### 2.2. Classification of Sparkling White Wine Style and Quality by MIR Spectroscopy

**Figure 4:**
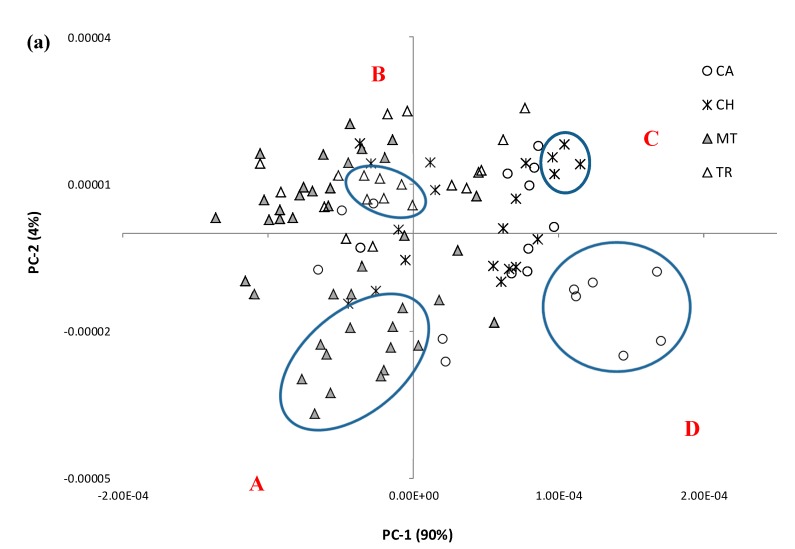

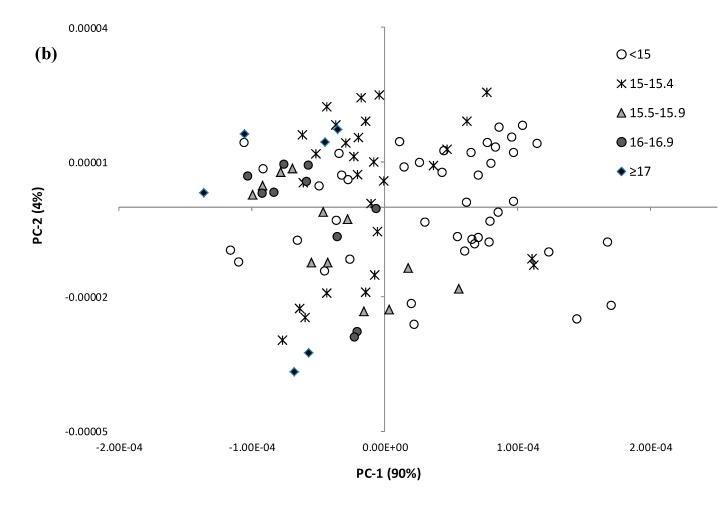
Score plots of the first two PC’s derived from the ‘fingerprint’ region (1500–900 cm^−1^) of MIR spectra from (degassed) sparkling white wines, labelled by (**a**) production method, CA = carbonated (*n* = 10), CH = Charmat (*n* = 10), MT = Methodé Traditionelle (*n* = 20) and TR = Transfer (*n* = 10); and by (**b**) quality scores. Sparkling wines located within the circled regions designated as **A**, **B**, **C** and **D** have sensory profiles displayed in Figure 6.

In 2012, the sparkling white wine segment held the lion share of Australian sparkling wine sales, both by value (54%) and by volume (61%) [18]. Sparkling white wines can also exhibit diverse sensory properties, depending on their method of production, which in turn influences quality and price. As such, the potential for ATR-MIR combined with PCA to classify sparkling white wines according to production method and/or quality was evaluated. Score plots displaying the first two PCs derived from the ‘fingerprint’ region of MIR spectra from 50 sparkling white wine samples (analyzed in duplicate, from two wine replicates) labelled according to production method and quality scores are shown as Figure 4a,b, respectively.

Differences were observed in the PCA score plot of sparkling white wines by method of production (Figure 4a). Carbonated and Charmat sparkling wines mostly clustered in the quadrants on the right; while Transfer and Methodé Traditionelle sparkling wines mostly clustered in the quadrants on the left. However, two carbonated wines and three Charmat wines overlapped with Transfer and Methodé Traditionelle wines. Furthermore, while wine replicates (*i.e.*, samples of the same wine, taken from different bottles) clustered closely together, they rarely overlapped, which indicates some bottle to bottle variation. The influence of production method on the style and sensory properties of sparkling wines is well established; carbonated and Charmat sparkling wines are typically fruit-driven styles, whereas Transfer and Methodé Traditionelle sparkling wines tend to exhibit complexity due to yeast autolysis and lees aging, post-secondary fermentation [2]; albeit sensory analysis indicated some carbonated and Charmat wines exhibited complexity, while some Transfer and Methodé Traditionelle wines displayed overt fruit aroma and flavor (data not shown). This may explain the overlap of some carbonated and Charmat wines, with Transfer and Methodé Traditionelle wines, and *vice versa*.

Classification of sparkling wines according to quality ratings was less evident, albeit a trend was observed (Figure 4b). With the exception of one wine, wines given a quality score of 15.5 or above (*n* = 14) were located in the left-hand quadrants. However, within these quadrants there were also ten wines with ratings between 15 and 15.4 and six wines given ratings below 15. The remaining 20 wines (with quality scores of 15.4 or less), were located in the right-hand quadrants. The difficulty in classifying sparkling wines based on quality scores may reflect the subjective nature of assessing quality. Assessments which rely on individual’s perceptions, even assessments by experts, *i.e.*, winemakers or wine show judges, are inevitably less well calibrated than analytical instruments. Furthermore, wines themselves can be deceiving. For instance, one of the carbonated sparkling wines was produced from an aged base wine and it therefore exhibited an unusual level of complexity, which may have contributed to its high quality score. Conversely, one Methodé Traditionelle wine exhibited noticeable volatile acidity (VA) and consequently received an especially low quality score. Whilst VA is ordinarily considered a winemaking fault, in this case, it was an intended to be indicative of a deliberate house style.

Partial least square (PLS) regression models were developed in order to determine any relationship between sparkling wine sensory attributes and MIR spectral data. The sensory profiles of each sparkling white wine were determined by descriptive analysis. A trained panel rated the intensity of 27 attributes, including a range of fruit and yeast-derived aromas and/or flavors, as well as sweetness, acidity and complexity. Nine of these attributes gave coefficients of determination (*i.e.*, R^2^) ≥ 0.50 (Table 2).

**Table 2:** Range, mean, standard deviation (SD) and cross validation statistics for sensory attributes in sparkling white wine samples analyzed by ATR-MIR spectroscopy.

Sensory Attribute	Range	Mean	SD	R^2^	SECV	PLS Terms
Sweetness P	2.3–8.3	4.81	1.37	0.72	0.73	4
Confection P	1.6–7.2	3.57	1.27	0.63	0.77	4
Tropical Fruit P	4.3–9.4	6.27	1.30	0.61	0.82	4
Meaty/Savoury P	1.3–6.0	2.86	1.05	0.59	0.68	3
Toasty P	2.4–8.0	4.83	1.29	0.57	0.86	4
Tropical Fruit A	3.6–8.8	5.20	1.48	0.56	1.00	4
Floral P	3.2–7.7	4.72	1.07	0.51	0.76	4
Floral A	2.6–8.4	4.83	1.54	0.51	1.08	4
Confection A	1.6–7.2	3.43	1.27	0.50	0.91	4

A = aroma, P = palate; SECV = standard error of cross validation.

The perception of *sweetness* gave the highest correlation, being R^2^ = 0.72; *i.e.*, *sweetness* ratings explained 72% of the variation within the PLS regression model. Ratings of *confection* and *tropical fruit* (on the palate) explained more than 60% of variation in the models; while >50% of variation was explained by intensity ratings for *tropical fruit*, *floral* and *confectionary* aromas and *meaty/savoury*, *toasty* and *floral* flavors on the palate. Previous studies have suggested correlations between spectral data and sensory attribute scores might result from co-linearity of compositional variables such as ethanol or residual sugar, or between wavelengths or other sensory properties [19,20]. It was interesting to note that the highest correlations were observed for the sensory attributes rated on the palate, rather than on the nose (*i.e.*, as aroma). Since most wine volatile compounds are present at low concentrations (*i.e.*, ng/L to mg/L levels), they are less likely to influence MIR spectra than more abundant wine constituents. Furthermore, when sensory panelists rate the intensity of aroma attributes, they may be preferentially evaluating the more volatile aroma compounds, *i.e.*, volatiles that are more abundant in the headspace of a wine glass. However, these volatiles may not be representative of a sample’s entire composition [20].

**Figure 5:**
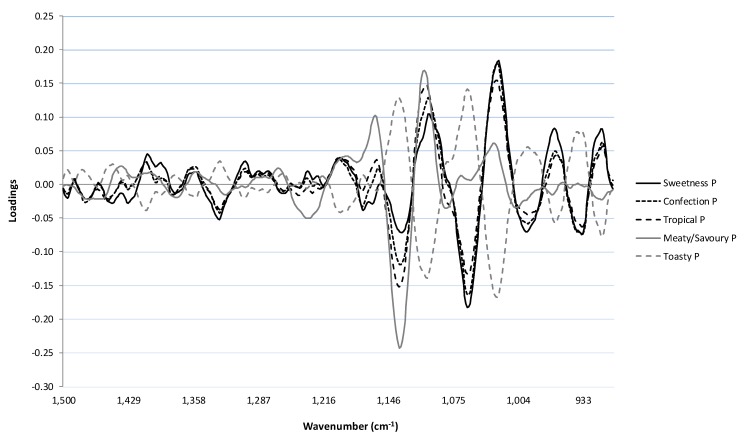
Optimal partial least squares regression coefficients derived from analysis of the MIR fingerprint spectra (1500–900 cm^−1^) against the top five correlated sensory attributes (*sweetness P*, *confection P*, *tropical P*, *meaty/savoury P* and *toasty P*) for the sparkling white wines. P denotes that the sensory attribute was rated on the palate.

The loadings for optimal PLS1 calibrations for the five most highly correlated sensory attributes (*i.e.*, *sweetness P*, *confectionary P*, *tropical fruit P*, *meaty/savoury P* and *toasty P*) are shown in Figure 5. The PLS loadings for *sweetness*, *confectionary* and *tropical fruit* followed similar patterns, with the highest positive loadings at 1025 and 1100 cm^−1^; *i.e.*, regions that had a large influence on the calibration models that were developed. As indicated above, these regions correspond to vibrational frequencies associated with the C–C and C–OH bonds of primary alcohols, glycerol and sugars [4,5,11,13,14,15]. Since the perception of sweetness is strongly influenced by both sugar and alcohol content, it is not surprisingly that regions corresponding to these compounds showed the greatest influence in the model. While the loadings for these three sensory attributes followed a similar pattern, there were differences in the size of loadings for some regions. For instance, the model for *tropical fruit* had less influence at 1025 cm^−1^ but more influence at 1100 cm^−1^, compared to the other two attributes. Furthermore, *sweetness* had higher positive loadings at 912 and 934 cm^−1^; regions associated with alkene and aromatic C–H vibrational frequencies [3]. There was also some variation in the region between 1420 and 1380 cm^−1^, which may be associated with stretching of C–H bonds from polysaccharides. Interestingly, the loadings for the *toasty* attribute had an inverse relationship to *sweetness*, *confectionary* and *tropical fruit*. Therefore, the wine constituents that contributed positively to the models for *sweetness*, *confectionary* and *tropical fruit* had a negative influence on the model for *toasty*. This suggests that the *toasty* model is driven by different wine constituents, particularly those corresponding to bonds with vibrational frequencies at 1060 and 1136 cm^−1^. Furthermore, the *toasty* attribute had positive loadings in the region of 1500–1400 cm^−1^, which could be related to volatile phenols, such as guaiacol and 4-methylguaiacol, which can impart *smoky* characters to wine [14]. However, this region has less influence in the model, as the loadings were relatively small. The *meaty/savoury* and *toasty* attributes are indicative of yeast autolysis or lees aging, whereas the ‘fresher’ characters of *sweetness*, *confectionary and tropical fruit* are likely to be grape or fermentation derived. It is therefore not surprising that the fresh characters appear to be driven by similar wine constituents.

**Figure 6:**
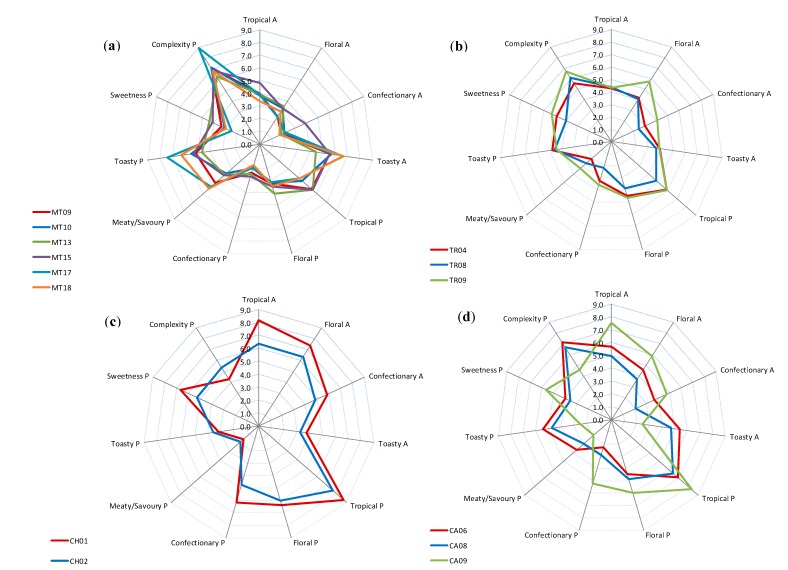
Sensory profiles of (**a**) Methodé Traditionelle (*n* = 6); (**b**) Transfer (*n* = 3); (**c**) Charmat (*n* = 2); and (**d**) carbonated (*n* = 3) sparkling wines that clustered in the circled regions A, B, C and D respectively, on the PCA score plot displayed as Figure 4a, (NB: circled regions comprise duplicates).

To further investigate the relationship between sparkling wine sensory attributes and MIR spectra, comparisons were made between the sensory profiles of selected sparkling wines that were clustered closely together; *i.e.*, those circled in regions designated as A, B, C and D in Figure 4a. The sensory profiles of Methodé Traditionelle (*n* = 6), Transfer (*n* = 3), Charmat (*n* = 2) and carbonated (*n* = 3) sparkling wines are shown in Figure 6a–d, respectively. For simplification, only sensory attributes which gave good correlations in the PLS regression models were included. Furthermore, the carbonated sparkling wine located within circled region B was excluded. In general, wines that clustered together in the PCA score plot (Figure 4a) were found to exhibit similar sensory profiles.

This was particularly evident for Methodé Traditionelle, Transfer and Charmat sparkling wines, but less so for carbonated sparkling wines, which exhibited the most style variation. The Methodé Traditionelle and Transfer wines exhibited considerable complexity, characterized by *meaty/savoury* and *toasty* characters, which typically result from yeast autolysis and lees-aging post-secondary fermentation. In some cases, sparkling wines are deliberately aged on yeast lees to enhance wine complexity and texture [2]. For example, production of MT17 involved >6 years lees aging; this almost certainly contributed to the high ratings of *toasty* and *complexity* on the palate (Figure 6a). In contrast, carbonated and Charmat sparkling wines predominantly exhibited fruit characters (Figure 6c,d). The sensory profile of CA09 was more representative of the carbonated sparkling wines studied; whereas CA06 and CA08 displayed unusually high complexity, due to extended ageing of their base wine. Although these three carbonated wines were clustered together on the PCA score plot (Figure 4a), their sensory profiles were less similar, suggesting different wine constituents influenced the MIR spectra and sensory properties. Since the PLS loadings suggested sugar and alcohol content both impacted the MIR spectra of sparkling wines, the sugar and alcohol concentrations of the wines depicted in Figure 6 were compared (Table 3) and clustering did indeed appear to be based on sugar and alcohol content. The Transfer sparkling wines (upper left quadrant) were high in both sugar and alcohol; Methodé Traditionelle sparkling wines (lower left quadrant) were low in sugar but higher in alcohol; Charmat sparkling wines (upper right quadrant) were high in sugar, but lower in alcohol; and carbonated sparkling wines (lower right quadrant) were low in both sugar and alcohol.

**Table 3:** Mean sugar and alcohol concentrations for those wines located in the circled regions A, B, C and D in each of the quadrants in the PCA score plot (Figure 4a).

**Upper Left Quadrant**	**Upper Right Quadrant**
Transfer (*n* = 3)	Charmat (*n* = 2)
RS = 12.2 g/L	RS = 17.3 g/L
Alcohol = 11.8% (abv)	Alcohol = 11.2% (abv)
**Lower Left Quadrant**	**Lower Right Quadrant**
Methodé Traditionelle (*n* = 6)	Carbonated (*n* = 2)
RS = 6.2 g/L	RS = 9.0 g/L
Alcohol = 11.9% (abv)	Alcohol = 10.5% (abv)

RS = residual sugar; abv = alcohol by volume.

The first PC therefore appears to be differentiating wines according to alcohol content, while the second PC gives separation based on sugar content. A similar trend was observed when the sugar and alcohol content of all sparkling wines was considered; on average: wines located within the upper left quadrant (*n* = 17) had 11.7 g/L of RS and 12.5% abv; wines within the upper right quadrant (*n* = 12) had 13.7 g/L of RS and 11.2% abv; wine from the lower left quadrant (*n* = 13 wines) had 8.2 g/L of RS and 12.1% abv; and wines situated in the lower right quadrant (*n* = 8) had 11.3 g/L of RS and 11.2% abv.

## 3. Experimental Section

### 3.1. Sample Preparation and Chemical Analysis of Sparkling Wines

Commercial Australian sparkling wines samples (*n* = 139), comprising vintage and non-vintage wines from a range of styles, *i.e.*, white (*n* = 50), rosé (*n* = 25), red (*n* = 25), Prosecco (*n* = 14) and Moscato (*n* = 25) wines, were sourced from wineries located across Australia. Prior to chemical analysis, sparkling wines were degassed, using a Sonorex Digitec DT 1028 F Ultrasonic bath (Bandelin Electronic GmbH & Co. KG, Berlin, Germany). Sparkling wines (approximately 50 mL) were placed in loosely capped centrifuge tubes and degassed for 30 min. The ultrasonic bath temperature was maintained below ambient temperature (by the addition of ice). Degassed wines were analyzed to determine pH and TA (as tartaric acid equivalents to an endpoint of pH 8.2) using a Crison CE95 Compact Titrator equipped with a Crison Sampler 15 autosampler (Crison Instruments, SA, Alella, Spain). Glucose and fructose (*i.e.*, residual sugar) were measured enzymatically (Boehringer-Mannheim/R-BioPharm, Darmstadt, Germany) using a liquid handling robot (CAS-3800, Corbett Robotics, Eight Mile Plain, Qld, Australia) and spectrophotometric plate reader (Infinite M200 Pro, Tecan, Grödig, Austria). Alcohol content (as % alcohol by volume) was measured with an alcolyzer (Anton Paar GmbH, Graz, Austria). Total phenolics was measured as the absorbance of wine at 280 nm, using either a GBC Cintra 40 or GBC Cintra 4040 UV-Visible spectrophotometer (GBC Scientific Equipment, Melbourne, Australia), according to methods reported previously [21].

### 3.2. Analysis of Sparkling Wines by Attenuated Total Reflectance-Mid-Infrared Spectroscopy

Sparkling wine samples (degassed; ca. 0.5 mL; in duplicate, taken from two different bottles) were scanned using a platinum diamond ATR single reflection sampling module cell mounted in a Bruker Alpha instrument (Bruker Optics GmbH, Ettlingen, Germany). The MIR spectra of samples were recorded on OPUS software (version 7, Bruker Optics) by taking the average of 32 scans at a resolution of 8 cm^−1^, acquired between 4000 and 400 cm^−1^, with a scanner velocity of 7.5 kHz and a background of 32 scans. Background reference spectra were recorded using air, every 4 samples.

### 3.3. Descriptive Analysis and Quality Rating of Sparkling White Wines

The sensory profiles and quality of the Australian sparkling white wines (*n* = 50), which comprised wines made from the four different production methods, *i.e.*, carbonation (*n* = 10), Charmat (*n* = 10), Transfer (*n* = 10) and Methodé Traditionelle (*n* = 20), were determined by descriptive analysis [22] and the 20 point scoring system used in Australian wine show judging [23], respectively. In each case, sparkling wines were chilled overnight at 5 °C prior to sensory evaluation. Wines were assigned random three digit codes and presented to panelists (as 30 mL samples) in coded Viticole XL5 (ISO standard) glasses, using randomized presentation orders.

Descriptive analysis was undertaken using a trained panel comprising ten University of Adelaide staff and students (all of whom had several years of wine tasting experience). Prior to formal evaluation, the panel underwent 12 h of training (*i.e.*, 6 × 2 h sessions held over three weeks), during which they identified appropriate descriptive terms and gained familiarity in recognizing and scoring the intensity of each attribute [23]. Formal evaluation sessions were then conducted twice weekly (over five weeks), with 15 sparkling wines presented at each session; to ensure all wines were assessed in triplicate. A total of 27 attributes were rated, including: *pome fruit* and *stonefruit* (as aroma only); *citrus*, *tropical* *fruit*, *floral*, *confection*, *meaty/savoury*, *mushroom*, *honey*, *yeasty*, *toasty*, *vanilla/caramel* and *aged/developed* (as aroma and palate); and *sweetness*, *acidity* and *complexity* (as palate only). Reference standards were presented at each session and panelists could refer to these at any time during evaluations. Panelists were presented with no more than four samples at a time; with breaks enforced between each sample (at least 45 s) and between each bracket (at least 3 min), to prevent sensory fatigue. Distilled water and crackers were provided as palate cleaners. Panelists assessed each sparkling wine in an isolated tasting booth at 22–23 °C and rated the intensity of each sensory attribute using a 15 cm unstructured line scale with anchor points of ‘low’ and ‘high’ placed at 10% and 90% on the scale, respectively; except for *sweetness* which used anchor points of ‘low’ and ‘medium’. Data acquisition was carried out using FIZZ software (Version 2.47b, Biosystemes, Couternon, France).

Quality ratings were undertaken with an expert panel comprising 19 winemakers and wine show judges, who met the criteria of ‘expert’ defined by Parr and coworkers [24]. Panelists assessed each sparkling wine (five of which were presented in duplicate) in an isolated tasting booth at 22–23 °C. Panelists were presented with five samples at a time and rated the quality of each sparkling wine using a 20 point scoring system. Quality scores from each judge were averaged to obtain a mean quality rating for each of the 50 sparkling white wines.

### 3.4. Data Analysis

Infrared spectra were exported from OPUS into The Unscrambler (Edition 10.2, CAMO ASA, Oslo, Norway) for chemometric analysis. Spectra were pre-processed using the second-derivative transformation, the Savitzky-Golay derivation and smoothing (20-point and 2nd-order filtering operation), to reduce baseline variation and enhance spectral features. PCA was performed on both the entire spectral range (4000 to 400 cm^−1^) and the MIR ‘fingerprint’ (*i.e.*, 1500 to 900 cm^−1^). PCA and PLS regression models were developed using cross validation. Sensory data were exported from FIZZ into Microsoft Excel and mean intensity ratings for each sensory attribute were determined for each sparkling white wine, using Senpaq software (version 5.01, Qi Statistics, Reading, UK).

## 4. Conclusions

This study demonstrated the capacity for ATR-MIR spectroscopy (combined with multivariate analysis) to broadly classify sparkling wines according to both style and production method. The results demonstrated qualitative compositional differences between wines that can be observed by MIR spectroscopy and used to distinguish wines, following PCA. Interestingly, discrimination was strongly influenced by the sugar and alcohol content of wines; *i.e.*, two of the more abundant wine constituents. However, some similarities in wine sensory profiles were also observed for wines that were closely clustered based on the ‘fingerprint’ region of their MIR spectra. ATR-MIR could therefore be used as a rapid method of screening large numbers of sparkling wines, so as to inform selection of a subset of wines for more detailed compositional and/or sensory analysis, by gas chromatography-mass spectrometry or descriptive analysis, for example.
